# The prevalence and factors associated with anxiety symptoms among resident physicians in Oman: a cross-sectional study

**DOI:** 10.1186/s43045-022-00214-z

**Published:** 2022-06-21

**Authors:** Basim AlJahwari, Ahmed AlKamli, Salim Al-Huseini, Moon Fai Chan, Badria AlMahroqi, Muna Al Saadoon, Aamal Ambusaidi, Aishwarya Ganesh, Samir Al-Adawi

**Affiliations:** 1 Family Medicine Residency Program, Oman Medical Specialty Board, Muscat, Oman; 2 Psychiatry Residency Program, Oman Medical Specialty Board, Muscat, Oman; 3 Department of Family Medicine and Public Health, College of Medicine & Health Sciences, Sultan Qaboos University, Muscat, Oman; 4 Directorate General of Health Services, Muscat Governorate, Ministry of Health, Muscat, Oman; 5 Department of Child Health, College of Medicine & Health Sciences, Sultan Qaboos University, Muscat, Oman; 6 Department of Behavioural Medicine, College of Medicine & Health Sciences, Sultan Qaboos University, Muscat, Oman

**Keywords:** Oman Medical Specialty Board (OMSB), Anxiety, Residents, Oman

## Abstract

**Background:**

Anxiety disorders are a significant factor associated with physician burnout and poor patient care, reported to have a significant frequency among the youth in the Middle East. However, to date, no study has explored the prevalence of anxiety among resident physicians in the Arabian Gulf country of Oman. This cross-sectional study, conducted among a random sample of residents affiliated with the Oman Medical Specialty Board, aimed to examine the frequency and factors associated with anxiety symptoms among them. Participants were asked to complete the *General Anxiety Disorder Assessment* (GAD-7) to assess anxiety, as well as a socio-demographic questionnaire.

**Results:**

In a total of 251 residents, the prevalence of anxiety was 14.7% (GAD-7 cut-off score ≥ 10). More than 60% of the respondents were female (68.9%). The age breakdown ranged from 25 to 30 years old (66.5%) and the majority were married (64.9%). More than 70% of respondents attended at least 5 shifts in their weekly schedule and received at least 5 on-call shifts from the hospital per week. Logistic regression showed that residents with chronic disease were 2.5 times (95% CI 1.36–4.72, *p* = 0.003) more likely to have anxiety than those without them. Those residents who did not exercise were 2.1 times (95% CI 1.04–4.46, *p* = 0.038) more likely to have anxiety than those who exercise often or regularly. Residents who received 6 or more on-calls from the hospital were 2.6 times (95% CI 1.35–5.25, *p* = 0.005) more likely to have anxiety than those who received 5 or fewer on-calls in a month.

**Conclusions:**

The factors seemingly responsible for anxiety symptoms in this sample of resident physicians are those that are typically associated with poor work-life balance and unhealthy lifestyles. Pending further scrutiny, these results could be used to lay the groundwork for the identification of those who will require more protracted help during their training in Oman and in other culturally similar Middle Eastern countries.

## Background

Mental health is an essential component of the World Health Organization’s (WHO) definition of health [1]. In the general population, anxiety disorders are a common mental health problem and are likely to be associated with higher morbidity and mortality [2]. The WHO has estimated that around 264 million people potentially live with anxiety disorders [3]. There is also evidence to suggest that anxiety symptoms are highly prevalent among healthcare professionals due to strenuous working conditions, which, in turn, precipitate occupational burnout and work-life imbalance [4].

Poor mental health outcomes have been increasingly reported in Arabian Gulf countries among students in tertiary education programs, especially in those training to be tomorrow’s doctors. This region is also the home to one of the youngest populations in the world, with a significant youth bulge in the population [5]. The budget allocated for education testifies to all the above factors. For example, 16% and 17% of the annual budgets are allocated to education in Saudi Arabia and the United Arab Emirates (UAE), respectively. Bahrain and Oman spend approximately 2% and 7%, respectively, of their total gross national product (GNP) on education [6].

In a recent narrative review, Al-Adawi et al. [7] reported that anxiety symptoms ranked fifth in the frequency of mental health problems among students in tertiary education in the Arabian Gulf countries. The highest was among (84.7%) the Saudi students, followed by Kuwaiti (63%) and Bahraini (51%) students. A specific anxiety disorder, social phobia, was reported to afflict 54% of the study sample in Oman. While these studies appear to elucidate the frequency of anxiety symptoms among “generic” students in various tertiary education programs, scant attention has been directed toward the well-being of the resident physician. Recent growth in medical services has also witnessed the exponential growth of medical schools in the Arabian Gulf countries. A significant proportion of post-graduate medical education is currently dispensed under the auspice of the Oman Medical Specialty Board (OMSB), adhering to the Accreditation Council for Graduate Medical Education-International (ACGME-I) [8, 9]. Due to the rising tide of poor mental health among healthcare workers, ACGME-I has stipulated measures to create cloistered training environments for the residents which, in turn, has the potential to mitigate issues related to burnout [10].

However, sparse data has been collected on the well-being of tomorrow’s doctors among those who fulfill the criteria of resident physicians under ACGME-I in Oman. Al-Shafaee et al. [11] explored the prevalence of abuse and mistreatment among first-year residents in Oman. Among the sampled residents, 96.6% support the contention that mistreatment exists in their educational setting, with verbal and academic abuses as the most common complaint, followed by sexual harassment and physical abuse. Additionally, Al Mukhaini et al. [12] reported that 37.3% of the resident physician sample of their study were “addicted to the internet” which, in turn, has strong associations with depressive symptoms. Additionally, the results of a study by Al-Houqani et al. [13] exploring the prevalence of depression among resident physicians in Oman reported that 28.8% exhibited depressive symptoms.

To date, no study has explored the prevalence of anxiety symptoms among resident physicians in Oman. Quek et al. [14] reported in a meta-analysis that the rate of anxiety among medical students in the Middle East and Asia exceeds those of their counterparts in the rest of the world, making it especially necessary to explore this statistical prevalence. Owing to the high levels of occupational stress intimately linked to their occupation, the presence of anxiety has the potential to hinder residents’ performance and affect the quality of healthcare provided [15, 16]. These issues will inevitably have negative effects on patient care and safety and, albeit indirectly, may trigger litigation and medical malpractice lawsuits. Therefore, this study has been embarked upon to examine the prevalence of anxiety symptoms among resident physicians and to analyze the possible socio-demographic and clinical predictors of anxiety among them.

## Methods

### Study design and setting

This cross-sectional analytical study was conducted on Oman Medical Specialty Board (OMSB) residents across different training programs, between January and April 2020. OMSB is an independent, ACGME-accredited educational body that sponsors and oversees graduate medical education (GME) programs in Oman. The inclusion criteria were (1) trainees or resident physicians officially enrolled with OMSB and (2) willing to consent to participate in the present study. Exclusion criteria included (1) non-resident physicians or allied disciplines; (2) students who were enrolled as part of optional internships, training or fellowships; and (3) those who did not consent to participate or did not complete the questionnaires.

### Data collection process

Data was collected using an electronic study survey. A self-administered questionnaire was sent to all residents of different specialties at different levels of residency in Oman. The participants were approached via their official OMSB email IDs. Individual program coordinators were contacted to confirm that the survey had been received by all residents. Of the 600 residents participating in OMSB residency programs, 100 were excluded from the study due to being on an extended leave for personal reasons, for completion of their residency training, or for master’s degrees and fellowships abroad. The electronic questionnaire was sent to 500 residents, and 251 responses were collected. The residency programs include medical subspecialties (i.e., family medicine, internal medicine, anesthesia, dermatology, emergency medicine, radiology, psychiatry, and pediatrics), surgical subspecialties (i.e., ENT, orthopedic, general surgery, ophthalmology, oral and maxillofacial surgery, obstetrics and gynecology), and diagnostic laboratory subspecialties (i.e., biochemistry, hematology, histopathology, and microbiology). For brevity and statistical analysis, these specialties were lumped into three groups: medical, surgical, or diagnostic, as detailed earlier [8].

The recruited participants were given information about the study and were informed that their participation would be completely anonymous and voluntary. A Declaration of Consent form was requested on the front page of the questionnaire, whereby the participant had to consent before proceeding with participating in the survey.

### Sampling method and sample sizes

A stratified random sampling procedure was adopted to ensure that the research sample would be representative. The algorithm for a randomization software was employed to fulfil the objective of the study. Thereafter, deemed representative participants were contacted to explain the objective of the present study and obtain electronic consent for participation. Previous studies have found a prevalence of anxiety in medical students in the range of 9.8 to 25.6% [17, 18]. With this assumption, and using the EPI Tools software [19], under 5% precision (margin error) at 95% confident intervals, the required samples would ideally range from 136 to 293, respectively.

### Outcome measures

The outcomes consisted of two parts: (i) socio-demographic and risk factors and (ii) quantification of anxiety symptoms. These are detailed below in tandem.

#### Socio-demographic and occupational factors

The first part of the survey solicited relevant socio-demographic information and questions probing about possible risk factors, with results shown in Table 1. Personal data was sought regarding gender, age, marital status, number of children, etc. The survey also inquired about where the participant’s residence or place of abode was, as OMSB attracts residents from different parts of the country (area = 309,501 km^2^). Oman has 11 administrative regions — Muscat, Dhofar, Musandam, Buraymi, Ad Dakhiliyah, North Batinah, South Batinah, South Sharqiyah, North Sharqiyah, Ad Dhahirah, and Al Wusta. The bulk of the population resides in the coastal region of the north of the country, known as Al Batinah coast. The place of residence was conveniently categorized as urban or rural. For brevity, the capital of Muscat, the largest metropolitan city along the coast overlooking the Arabian Sea, was categorized as “urban” and the rest were categorized as “rural.”

**Table 1:** Socio-demographic and occupational variables of the study samples (*n* = 251)

Variable	*n* (%)
**Gender**	
Female	173 (68.9)
Male	78 (31.1)
**Age (years)**	
Below 25	2 (0.8)
25 to 30	167 (66.5)
30 to 35	77 (30.7)
Above 35	5 (2.0)
**Marital status**	
Single	87 (34.7)
Widowed	1 (0.4)
Married	163 (64.9)
**Number of children**	
0	126 (50.2)
1	67 (26.7)
2	39 (15.5)
3 and above	19 (7.6)
**Place of residence**	
Rural	66(26.3)
Urban	185 (73.7)
**Domestic help**	
No	131 (52.2)
Yes	120 (47.8)
**Family support**	
No	23 (9.2)
Yes	228 (90.8)
**Chronic disease**	
Yes	26 (10.4)
No	225 (89.6)
**Doing exercise**	
No	126 (50.2)
Often	106 (42.2)
Regular	19 (7.6)
**Residency program**	
Medical	213 (84.9)
Surgical	25 (10.0)
Diagnostic	13 (5.1)
**Year of study**	
1	60 (23.9)
2	54 (21.5)
3	62 (24.7)
4	57 (22.7)
5	14 (5.6)
6	4 (1.6)
**Number of examinations per year**	
1	62 (24.7)
2	45 (17.9)
3	13 (5.2)
4 and above	131 (52.2)
**Examination results**	
Fail	6 (2.4)
Pass	245 (97.6)
**Number of shifts in the hospital scheduled per week**	
4 and below	71 (28.3)
5	109 (43.4)
6 and above	71 (28.3)
**Number of on-calls from the hospital per week**^**a**^	
3 and below	39 (15.7)
4	49 (19.8)
5	98 (39.5)
6	60 (24.2)
7 and above	2 (0.8)
**Anxiety (GAD-7)**	
Yes (GAD-7 total score ≥10)	37 (*14.7*)
No (GAD-7 total score < 10)	214 (85.3)

*GAD-7*, Generalized Anxiety Disorder Scale, the total score ranged from 0 to 21; ^a^3 missing

In addition to personal data and place of abode, this study solicited factors that may affect anxiety positively or negatively. Participants were also asked whether they felt as though they received adequate support from their homes and from the residency training program. They were also asked about the number of shifts they had scheduled per week, and how many times they received on-calls from the hospital per week. Such occupational variables were partly employed for the fact that they reflect the ACGME-I charter that aims to improve the well-being of residents, and partly to reflect specific demographic trends in Oman that befits a demographic population in transition [20, 21].

#### Generalized Anxiety Disorder (GAD-7) scale

The study utilized a widely-used checklist, the Generalized Anxiety Disorder (GAD-7) assessment, to find the frequency of the symptoms of anxiety in the sample [22]. GAD is a seven-item screening tool with items derived from DSM-IV criteria for generalized anxiety disorder. In reference to the previous two weeks, the participant responded on a 4-point Likert scale (0 = “not at all,” 1 = “several days,” 2 = “over half of the days,” 3 = “nearly every day”). GAD-7 has been validated in various languages and ethnic groups [23]. The Arabic version has been shown to have parallel psychometric properties to the original version [24]. The score for GAD-7 ranged from 0 to 21. The cut-off of >10 has been widely considered to differentiate those with anxiety and those without [22]. For the present cohort, the internal consistency of the GAD-7 was adequate (Cronbach’s *α* = 0.83).

### Statistical analysis

Descriptive statistics, including frequency and percentage calculation, were used to explore the profile of the participants according to their socio-demographic and occupational information. The anxiety status as a dependent variable determined by the GAD-7 total score and socio-demographic and occupational data were independent variables. First, the univariate analysis included chi-square, and Fisher’s exact tests were used to identify factors associated with anxiety. Then, those factors that showed significance at the 5% alpha level in the univariate analysis were included in the logistic regression (backward Wald method) to further analyze the risk factors associated with anxiety. All analyses were conducted with SPSS 27.0 (IBM SPSS Inc. Chicago, IL, USA) and set at a 5% significance level.

### Ethics approval

This work was approved by the research ethics committee of the Oman Medical Specialty Board, Muscat, Oman (REC /01/2019). Informed consent was collected from all participants. The study was conducted following the Declaration of Helsinki and the American Psychological Association regarding ethical human research, concerning confidentiality, privacy, and data management.

## Results

A total of 251 residents (response rate = 50.2%) participated in this study. Table 1 shows the socio-demographic and occupational characteristics of the participants. More than 60% of them were females (68.9%), were between 25 to 30 years old (66.5%), and were married (64.9%). The majority of them were living in urban areas (73.7%), more than half of them did not have domestic help (52.2%), and the majority were supported well by their family members (90.8%). Around 10% of residents had a chronic disease, and half of them (50.2%) do not regularly exercise. A majority of residents enrolled in the residency program were specializing in medical programs (84.9%), followed by surgical (10.0%) and diagnostic programs (5.1%). The study participants comprised a near-equal proportion of around 20% each from year 1 to year 4 of residency, with a lesser number of participants drawn from year 5 (5.6%) and year 6 (1.6%). 52.2% of residents had 4 or more examinations per year, and only a small percentage (2.4%) failed their examinations. More than 70% of them had at least 5 shifts scheduled per week and received at least 5 on-calls from the hospital per week. According to the total score of the GAD-7 and the cut-off utilized for the purposes of this study, the prevalence rate of anxiety is 14.7% (*n* = 37).

In Table 2, univariate analysis showed that chronic disease (*p* = 0.006), doing exercise (*p* = 0.022), year of study (*p* = 0.038), higher number of shifts (*p* = 0.010), and higher number of on-calls received (*p* = 0.003) from the hospital were significant factors associated with anxiety. No significant differences were seen between anxiety and the other variables. All the significant factors in univariate analysis were used in the logistic regression for further analysis.

**Table 2:** Univariate analysis for anxiety in the association of socio-demographic and occupational variables

Variable	Anxiety (GAD-7)		*P*-value
	Yes (*n* = 37)	No (*n* = 214)	
	*n* (%)	*n* (%)	
**Gender**			
Female	29 (78.4)	144 (67.3)	0.178
Male	8 (21.6)	70 (32.7)	
**Age (years)**			
Below 25	0 (0.0)	2 (0.9)	0.915
25 to 30	24 (64.9)	143 (66.8)	
31 to 35	12 (32.4)	65 (30.4)	
36+	1 (2.7)	4 (1.9)	
**Marital status**			
Not married	16 (43.2)	72 (33.6)	0.259
Married	21 (56.8)	142 (66.4)	
**No. of children**			
0	20 (54.1)	106 (49.5)	0.594
1	9 (24.3)	58 (27.1)	
2	7 (18.9)	32 (15.0)	
3 and above	1 (2.7)	18 (8.4)	
**Place of stay**			
Rural	7 (18.9)	59 (27.6)	0.317^
Urban	30 (81.1)	155 (72.4)	
**Domestic help**			
No	21 (56.8)	110 (51.4)	0.547
Yes	16 (43.2)	104 (48.6)	
**Family support**			
No	6 (16.2)	17 (7.9)	0.122^
Yes	31 (83.8)	197 (92.1)	
**Chronic disease**			
Yes	9 (24.3)	17 (7.9)	0.006^
No	28 (75.7)	197 (92.1)	
**Doing exercise**			
No	25 (67.6)	101 (47.2)	0.022
Yes (often/regular)	12 (32.4)	113 (52.8)	
**Residency programs**			
Medical	31 (83.8)	182 (85.0)	0.138
Surgical	6 (16.2)	19 (8.9)	
Diagnostic	0 (0.0)	13 (6.1)	
**Year of study**			
3rd and above	26 (70.3)	111 (51.9)	0.038
1st and 2nd	11 (29.7)	103 (48.1)	
**No. of exams**			
1	9 (24.3)	53 (24.8)	0.991
2	6 (16.2)	39 (18.2)	
3	2 (5.4)	11 (5.1)	
4 and above	20 (54.1)	111 (51.9)	
**Exam result**			
Fail	2 (5.4)	4 (1.9)	0.216^
Pass	35 (94.5)	210 (98.1)	
**Number of shifts per week**			
6 and above	17 (45.9)	54 (25.2)	0.010
5 and below	20 (54.1)	160 (74.8)	
**Number of on-calls from the hospital per week**^**a**^			
6 and above	17 (45.9)	45 (21.3)	0.003
5 and below	20 (54.1)	166 (78.7)	

*GAD-7* Generalized Anxiety Disorder Scale, the total score ranged from 0 to 21, anxiety: GAD-7 total score ≥10; no anxiety: GAD-7 total score < 10; ^a^3 missing; ^Fisher’s exact test

In Table 3, five factors (chronic disease, exercise, year of study, number of shifts, and number of received on-calls from the hospital) were included in the logistic analysis for further analysis. Results showed that chronic disease, exercise, and a higher number of on-calls received from the hospital were significant determinants of anxiety (Table 3). The model has a good-fit result (chi-square = 3.43, *p* = 0.331) with an adjusted Cox and Shell *R*^2^ of 0.464. It has an acceptable sensitivity (54.1%), good specificity (75.4%), and overall predicting power (72.2%). Results showed that residents with chronic disease were 2.5 times (OR = 2.54, 95% CI 1.36–4.72, *p* = 0.003) more likely to have anxiety than those without chronic disease. Those residents who do not exercise were 2.1 times (OR = 2.16, 95% CI 1.04–4.46, *p* = 0.038) more likely to have anxiety than those who are regularly exercising. Regarding occupational factors, residents who received 6 and above on-calls from the hospital were 2.6 times (OR = 2.66, 95% CI 1.35–5.25, *p* = 0.005) more likely to have anxiety than those who received 5 and below on-calls from the hospital.

**Table 3:** Logistic analysis for anxiety in the association of socio-demographic and occupational variables

Variable	OR (95% CI of OR)	*P*-value
Chronic disease		
Yes	2.54 (1.36–4.72)	0.003
No^b^		
Doing exercise		
No	2.16 (1.04–4.46)	0.038
Yes (often/regular)^b^		
Number of on-calls from the hospital per week^a^		
6 and above	2.66 (1.35–5.25)	0.005
5 and below^b^		

*OR* odds ratio, *CI* confidence intervals; logistic (Backward Ward): Hosmer and Lemeshow test (chi-square = 3.43, *p* = 0.331), Cox and Shell *R* square = 0.464, sensitivity = 54.1%, specificity = 75.4%, overall = 72.2%; ^a^3 missing; ^b^reference point

## Discussion

Stress has been suggested to be at the top of the list of mental health issues among students enrolled in tertiary education programs in the Arabian Gulf [7]. This is also the trend worldwide among college students, who often complain of being stressed, burned out, or exposed to adverse experiences [25]. A Canadian campus survey reported a 30% prevalence of perceived stress among college-going students [26]. Recently, in Malaysia, perceived stress was present in 37.7% of the college-going population, while in India, the corresponding percentage was 42.5%, and in Pakistan, 58.9% [27–29]. In a recent review among Arabian Gulf students, perceived stress and feelings of being burned out were reported among 92.5%, 96.3%, and 89.2% of Saudi, Bahraini, and Qatari students, respectively [7].

As the concept of stress is conceptualized as “any type of change that causes physical, emotional, or psychological strain” [30], there is a need to examine those distresses among students in tertiary education using diagnostic tools that parallel the available psychiatric nosology, so that their severity and possible intervention methods could be contemplated.

With this background, the present study was embarked upon to examine the frequency of anxiety symptoms by using the Generalized Anxiety Disorder (GAD-7) assessment among resident physicians in Oman. The prevalence of anxiety symptoms in the present study among resident physicians was *14.7%*.

This figure appears to be in the lower ranges compared to those established in other similar studies within the Arabian Gulf countries that have employed various instruments such as the anxiety subscale of Hospital and Depression Scale (HADS), the anxiety subscale of Depression Anxiety Stress Scales (DASS-21), Zung Self-Rating Anxiety Scale (ZSAS) and Becks Anxiety Inventory (BAI), as well as the General Anxiety Disorder (GAD-7) used in the present study. In Saudi Arabia, the frequency of anxiety symptoms in medical students has been reported to range from 31.7 to 34.9% [31, 32]. In Bahrain, the rate of anxiety symptoms in medical and nursing students was found to range from 9.7 to 51% [33, 34], while in the UAE, the rate ranges from 22.3 to 63.1% [35, 36]. It is worthwhile to note that these studies have recruited generic students from various tertiary education programs, rather than samples specifically composed of medical trainees or resident physicians. However, studies have suggested that resident physicians are more prone to suffer from anxiety symptoms compared to other trainees or practitioners [37]. Other studies have indicated that poor coping mechanisms follow a cumulative pattern, wherein those who reported poor coping upon initiating medical training are likely to have persistently poor coping strategies throughout their training and practice [38, 39]. With this theory in mind, it is important to note that perceived stress among medical students in Oman, who are in the process of training to become future residents, has a significant prevalence rate of 51.4% [40].

The aforementioned discussion on the present result within the extant literature suggests that anxiety symptoms manifest in a complex way among tomorrow’s doctors. Therefore, exploring psychosocial characteristics and factors associated with anxiety symptoms have the potential to shed light on the development of poor coping strategies and the trajectories of anxiety symptoms. In a narrative review on associated factors for students in tertiary education including medical trainees in the Arabian Gulf countries, it was noted that poor mental health problems were strongly associated with substance misuse, high screen time, course difficulties, and resultant poor academic performance and sleep problems [41]. In Brazilian populations, Carneiro Monteiro et al. [42] have reported that factors associated with stress and distress among resident physicians included one’s age, the nature of their relationships with mentors in their respective institutions, and “home issues,” such as relationships with significant others. From Lebanon, Zarzour et al. [43] have reported that anxiety symptoms were strongly associated with being female, belonging to a younger age group in the sample cohort, and living with the elderly. In a multicenter study reported from China, Bai et al. [44] reported factors associated with anxiety symptoms among resident physicians included disturbances in sleep-wake cycles. The participants were also marked with elevated scores in indices of burnout such as emotional exhaustion, depersonalization, and reduced personal accomplishment. A systematic review by Dubale et al. [45] reported that perceived occupational stress among healthcare providers in sub-Saharan Africa was strongly related to organizational lack of support and “toxic” work environments, leading to caustic work relationships.

As most studies have examined factors associated with adversity, Mascaro et al. [46] have examined factors associated with well-being among healthcare trainees (resident physicians and physician assistant trainees). The study indicated that factors that lead to “flourishing” among trainees were common among those who exercised more frequently and accrued less abstention from training. The present study also explored the association between anxiety and exercise, or more specifically, the lack of exercise, which was found to be significantly related to the development of anxiety symptoms. Only 32.4% of the present resident physician were exercising regularly. In the regression analysis, those who did not exercise were 2.1 times (95% CI 1.04 – 4.46, *p* = 0.038) more likely to have anxiety than those who exercise often or regularly. Nutting et al. [47] have conducted an intervention trial in which, after 10 months of physical training, the majority of the participants showed a significant reduction in indices of anxiety symptoms. To date, most of the effort directed toward stress reduction has been geared toward psychological interventions or institutional changes to accommodate the need of trainees [48]. The aforementioned preliminary studies of the role of exercises are worthwhile to consider in Oman, where there is a lack of professional skill in dispensing western-based psychological intervention. In the general population, physical exercises are increasingly recognized as a “panacea” for many medical ailments [49, 50]. This region has also been equated with a high preponderance of sedentary lifestyles [51]. It is possible that ecological factors, such as high temperatures, and social factors such as discomfort among women for exercising in public, might contribute to the lack of practicing regular exercise among the resident physician sample of Oman. Many studies have reported the positive effects of physical activity on the management of anxiety disorders and other mental health issues [52, 53]. More studies are therefore warranted to analyze the effects of exercise on the mental health of Omani residents, keeping in mind the potential benefits as well as the socio-demographic background of the region.

Among the current studies’ sample of 251, 68.2% (*n* = 173) of the study were female residents and 31% (*n* = 78) were male residents. The increased prevalence of female physicians experiencing greater levels of anxiety and job stress than their male counterparts seems to be in agreement with existing literature [54]. The high number of female participants in this study is not necessarily an artifact of the recruitment process, but factors likely to be linked to the third phase of demography in transition, which has resulted in increased entrance of women into the labor force [21]. When Oman is gleaned via the prism of epidemiologic demographic transition theory, recent development has resulted in the country achieving the third phase of demographic transition [21]. The prevailing trend observed in the Omani population is the decline of the birth rates and mortality rates, as well as the increased plasticity of life span and expansion in women’s education. This has triggered the empowerment of women in the labor force and an erosion of traditional modes of living, wherein women were previously limited to the domestic sphere only [55]. In Oman, more women are currently enrolled in medical school and graduating as doctors than men, and the percentage of female general practitioners is exponentially rising [56].

While the majority of participants were married, 34.7% of them were single. While there is no significant association between the number of children and anxiety status in the present sample, the majority appear to have one (40.9%) to two (23.8%) children. While being single in the traditionalist society of Oman is generally culturally under-valued, this study indicates that single resident physicians exhibited less anxiety than married physicians, at 43.2% and 56.8%, respectively. However, this link was not proven to be statistically significant (*p* < 0.259). It is still important to consider how married resident physicians juggling their professional careers and their traditional roles in the family can cause increased levels of stress and anxiety related to poor work-life balance [57].

Work-life balance is increasingly recognized to contribute to poor coping among healthcare workers, because of working in shift-based schedules and the nature of the strenuous workload [4]. Related to this, residents who received 6 or more on-calls from the hospital were 2.6 times more likely to exhibit anxiety symptoms (95% CI 1.35–5.25, *p* = 0.005) than those who received fewer on-calls. In existing literature, there is consensus that being “on-call” leads to more fatigue and the disturbance of a healthy circadian rhythm [58]. Tucker et al. [59] have conducted a study to examine the factors leading to fatigue and well-being among junior doctors working on different shifts. They reported that being on frequent on-calls was associated with increased work-life imbalance and indices of psychological strain. Rodriguez-Jareño et al. [60] conducted a systematic review on the adverse effects of not adhering to the provision known as the European Working Time Directive (EWTD) on physicians. They reported that long working hours, which naturally entail frequent on-calls, increased risk factors for accidental injuries and road traffic accidents. The negative impact of emergency on-calls and erratic work schedules on the physical and psychological well-being of physicians has been widely reported in existing literature from various settings [61–64].

Another domain that emerged to be significant in the regression analysis is the presence of chronic disease. While it might be expected that physicians’ health status would be free from persistent and pervasive chronic diseases in order to maintain the rigorous lifestyle required, this study nevertheless found a significant association between chronic disease and anxiety symptoms. Among those with chronic illness, 24.3% exhibited a high level of anxiety symptoms, therefore being 2.5 times more likely to develop anxiety symptoms (95% CI 1.36–4.72, *p* = 0.003) than those without a chronic disease. It has been widely established that the presence of chronic illness tends to create a “psychological burden” and, conversely, the presence of anxiety symptoms tends to further dent the chronic illness [65]. The impact of chronic illness on the presentation of anxiety among physicians has also been reported in other samples around the world [66, 67]. Since chronic illness was found to be a significant factor associated with anxiety, future studies should properly define and specify what constitutes chronic illness and how specific conditions relate to the psychological well-being of resident physicians. Related to this, mechanisms should be present in the methodology of future studies to rule out the presence of medical conditions that mimic or present as anxiety, such as neuroendocrinal conditions like hyperthyroidism and Cushing’s disease, and cardiac diseases like mitral valve prolapse [68].

This study is the first cross-sectional study investigating the prevalence of anxiety among resident physicians in Oman but has some *limitations* that are worth noting. *Firstly,* as is often the case, cross-sectional studies are not capable of establishing temporal relationships. *Secondly*, the survey study was conducted during the COVID-19 pandemic. Therefore, the possibility remains that the observed magnitude of anxiety symptoms is related to this period of tribulation caused by the pandemic, rather than the specific situation of being a resident. However, notwithstanding such a view, the prevalence rate of 14.7% appears to be in the lower range even though anxiety symptoms have been reported to have spiked during the COVID-19 pandemic among healthcare workers [69]. *Third*, although the present study’s response rate of 50% may echo the international trend among other similar studies that have used online surveys [70], it still stands that the present sample included only half of the residents of this program. *Fourth,* this study did not have mechanisms to rule out chronic illnesses that are known to cause anxiety as a part of their symptomology, resulting in a potential confounding factor. *Last* but not least, it is not clear whether the reported associated factors of the current study were specific to those who responded, resulting in possible bias. Further analysis on this issue is therefore warranted.

## Conclusions

The findings of the study indicate that anxiety is common among resident physicians of Oman, but the frequency appears to be lower when compared to the trend observed among resident physicians of other countries. It is evident from the study that various factors are associated with anxiety among them. The factors seemingly responsible for anxiety symptoms in this sample of resident physicians are typically those that are associated with poor work-life balance and unhealthy lifestyles. Preventive and treatment strategies are highly recommended by increasing the residents’ awareness about their current mental health, as well as educating them about positive coping strategies that can improve their personal and professional development. These findings can be used to design appropriate and systematic interventions and programs to help residents at risk of anxiety. Robust support and increased psychological assessment and monitoring must be taken seriously to avoid higher prevalence rates of poor mental health in the doctors of the future.

## Data Availability

This is a research article and all data generated or analyzed during this study are included in this published article.

## Abbreviations

WHO: World Health Organization
UAE: United Arab Emirates
OMSB: Oman Medical Specialty Board
ACGME-I: Accreditation Council for Graduate Medical Education-International
GAD-7: Generalized Anxiety Disorder assessment
OR: Odds ratio
CI: Confidence interval
HADS: Hospital and Depression Scale
DASS-21: Depression Anxiety Stress Scales
BAI: Becks Anxiety Inventory
COVID-19: Coronavirus disease of 2019
